# Complete Genome Sequence of Multidrug-Resistant Clinical Isolate *Mycobacterium tuberculosis* 187.0, Used To Study the Effect of Drug Susceptibility Reversion by the New Medicinal Drug FS-1

**DOI:** 10.1128/genomeA.01272-15

**Published:** 2015-11-05

**Authors:** Aleksandr I. Ilin, Murat E. Kulmanov, Ilya S. Korotetskiy, Gulshara K. Akhmetova, Marina V. Lankina, Sergey V. Shvidko, Oleg N. Reva

**Affiliations:** a Scientific Center for Anti-Infectious Drugs (SCAID), Almaty, Kazakhstan; b Center of Bioinformatics and Computational Biology (CBCB), Department of Biochemistry, University of Pretoria, Pretoria, South Africa

## Abstract

Complete genome sequence of the multidrug-resistant clinical isolate *Mycobacterium tuberculosis* SCAID 187.0 containing several drug-resistance mutations is presented. This strain is used in experiments to study genomic and population changes leading to reversion of susceptibility to the 1st line anti-tuberculosis (TB) drugs under the influence of a new medicinal drug FS-1.

## GENOME ANNOUNCEMENT

Tuberculosis (TB) again threatens mankind due to the distribution of multidrug-resistant (MDR) strains. Development of new antibiotics is a challenge for pharmaceutical companies resulting in the fact that not one anti-tuberculosis drug has been registered for the last 35 years (1, 2). A new medicinal drug FS-1 (3) has passed clinical trials and was registered as a new drug against MDR tuberculosis in Kazakhstan. A specific therapeutic activity of FS-1 is that it can reverse susceptibility to antibiotics traditionally used for tuberculosis treatment in patients with MDR infection. Reversion of drug susceptibility is considered to be a prospective way of combating the growing problem of drug resistance (4). The aim of this research was to create the grounds for further study of the drug susceptibility reversion phenomenon by sequencing the drug-resistant clinical strain of *Mycobacterium tuberculosis* (MDR-TB) showing a clear positive response to FS-1 in laboratory conditions.

The strain SCAID 187.0 was isolated from sputum of a patient with MDR-TB during the 2nd phase of the clinical trial of FS-1. The strain was isolated before intake of the first dose of FS-1. A drug susceptibility test on solid and liquid media showed resistance to the 1st line of anti-TB-drugs and to some anti-TB-drugs from the 2nd line. The strain was used in laboratory experiments on guinea pigs to study the reversion of drug susceptibility. In total, 15 samples of the strain were prepared for sequencing from the frozen stock culture including the strain isolated from the patient after 180 days of treatment with FS-1 and samples collected from animals used in experiment at different conditions: (i) without anti-TB treatment, (ii) treated with the standard set of anti-TB-drugs (pyrazinamide, 100 mg/kg; cycloserine, protionamide, and capreomycin, 20.0 mg/kg; and amikacin, 50.0 mg/kg), and (iii) treated with anti-TB drugs supplemented with FS-1 in concentrations of 2.5 and 4.0 mg/kg. Fourteen samples of genomic DNA were sequenced in Macrogen (South Korea) using a paired-end Illumina Hiseq 2000; one sample of the stock culture was sequenced by an Ion Torrent PGM in SCAID. In total, 166,252,936 DNA reads were generated. The reads generated from the stock culture and samples from animals treated only with antibiotics were used for assembly of the complete genome sequence. Other samples were used for gap closure and variant calling. *De novo* and reference based assembly were performed by CLC Genomics Workbench 7.0.3. Genomes *M. tuberculosis* H37Rv (NC_000962) and *M. tuberculosis* NITR203 (NC_021054.1) were used as references.

The assembled genome of SCAID 187.0 is 4,379,515 bp long and C+G-content is 65.6%. Genome annotation was performed by using the RAST server (5) and corrected manually. In total, 4,097 protein coding genes and 45 genes for tRNA were found. The pattern of single nucleotide polymorphisms (SNPs) of SCAID 187.0 was characteristic for the clade Beijing. A search through polymorphic sites identified several canonical antibiotic resistance mutations (6): S95T in GyrA (7), K43R in RpsL (8), M306I in EmbB (9), and S450L in RpoB (10) causing fluoroquinolone, streptomycin, ethambutol, and rifampin resistance, respectively.

### Nucleotide sequence accession number.

The complete genome sequence is available in GenBank under accession no. CP012506. The BioSample no. is SAMN03921880 and the BioProject no. is PRJNA291131.
